# DCJ-Indel sorting revisited

**DOI:** 10.1186/1748-7188-8-6

**Published:** 2013-03-01

**Authors:** Phillip EC Compeau

**Affiliations:** 1 Department of Mathematics, UC San Diego, 9500 Gilman Drive 0112, San Diego, CA 92093, United States

**Keywords:** Genome rearrangements, DCJ, Indels, Sorting, Solution space

## Abstract

**Background:**

The introduction of the double cut and join operation (DCJ) caused a flurry of research into the study of multichromosomal rearrangements. However, little of this work has incorporated indels (i.e., insertions and deletions of chromosomes and chromosomal intervals) into the calculation of genomic distance functions, with the exception of Braga et al., who provided a linear time algorithm for the problem of DCJ-indel sorting. Although their algorithm only takes linear time, its derivation is lengthy and depends on a large number of possible cases.

**Results:**

We note the simple idea that a deletion of a chromosomal interval can be viewed as a DCJ that creates a new circular chromosome. This framework will allow us to amortize indels as DCJs, which in turn permits the application of the classical breakpoint graph to obtain a simplified indel model that still solves the problem of DCJ-indel sorting in linear time via a more concise formulation that relies on the simpler problem of DCJ sorting. Furthermore, we can extend this result to fully characterize the solution space of DCJ-indel sorting.

**Conclusions:**

Encoding indels as DCJ operations offers a new insight into why the problem of DCJ-indel sorting is not ultimately any more difficult than that of sorting by DCJs alone. There is still room for research in this area, most notably the problem of sorting when the cost of indels is allowed to vary with respect to the cost of a DCJ and we demand a minimum cost transformation of one genome into another.

## Background

In the simplest terms, DNA may mutate in two fundamentally different ways. On the one hand, single-nucleotide polymorphisms alter the base at a single position of the nucleic acid polymer; on the other hand, huge mutations called chromosomal rearrangements can move around, duplicate, insert, or delete huge blocks of DNA, often from one chromosome to another.

Chromosomal rearrangements were first observed by Dobzhansky and Sturtevant in 1938 ([1]), but extensive efforts to quantify their study did not take off until the early 1990s. In the last two decades, a number of discrete genomic models have been proposed and studied (see [2] for an overview of the combinatorics of genome rearrangements).

Having selected a genomic model and a collection of genome operations to consider, the standard algorithmic problem is the computation of the distance between two genomes *π *and *Γ*, or the minimum number of allowable operations required to transform *π *into *Γ*; the more difficult problem of sorting demands the operations themselves. The first historical example of such a discrete genomic distance is the prefix reversal distance for permutations (which model the order of genes along a single linear chromosome), introduced in [3] and bounded in [4-6]. The computation of prefix reversal distance has been proposed to be *NP*-Hard (see [7]).

More recent research has moved past permutations and toward multichromosomal genomic models that incorporate both linear and circular chromosomes. One of these models, which we will study in this paper, models the chromosomes of a genome with paths and cycles in a graph. For this model, the double cut and join operation (DCJ) was introduced in [8] and incorporates segment reversals with a number of other operations. Interestingly, a linear time greedy algorithm exists for DCJ sorting two genomes having equal gene content (see [9]).

The incorporation of insertions and deletions of chromosomes and chromosomal intervals (collectively called *indels*) into DCJ distance was discussed in [10] and quantified rigorously in [11]. The latter authors provided a linear time algorithm for the associated problem of DCJ-indel sorting, which gives a minimum collection of DCJ and indel operations required to transform one genome into another. Yet their argument is case-ridden, and so in this paper, which builds upon [12], we wish to provide a much simpler presentation of DCJ-indel sorting that still yields a linear-time solution to the problem.

## Main text

### Preliminaries

**Figure 1:**
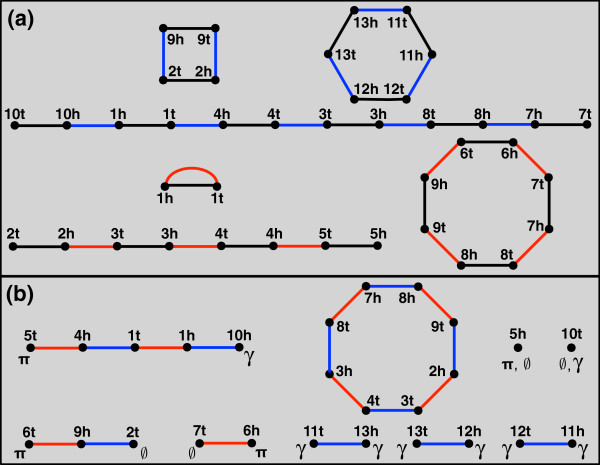
**Two Genomes and their Breakpoint Graph. (a) **Genomes *π *and *Γ *on a collection of 12 genes. We use “h” and “t” to denote the head and tail of a gene. *π *is drawn with blue adjacencies, and *Γ *is drawn with red adjacencies. **(b) **The breakpoint graph of *π *and *Γ*. We have labeled the endpoint *v *of a path with *π* if *v *is *π*-open, with *Γ *if *v *is *Γ*-open, and with *∅ *if *v* is a telomere of at least one genome.

Say that we are given a perfect matching on 2*N *labeled vertices V, forming a set G of *N *edges called genes; the vertices of each gene form its head and tail. We define a genome *π *as the edge-disjoint union of two matchings. The genes of *π*, denoted *g*(*π*), form a matching on V such that g(π)⊆G; the adjacencies of *π*, denoted *a*(*π*), form a matching on *V*(*g*(*π*)). We color the genes of *π* black and the adjacencies of *π *blue (see Figure 1(a)).

A consequence of these definitions is that *π *comprises a disjoint collection of paths and cycles, where each connected component alternates between black genes and blue adjacencies. Each component of *π *is called a *chromosome*; paths (cycles) of *π *define *linear *(*circular*) chromosomes of *π*. The endpoint *v *of a path in *π *is called a telomere of *π*; *v *is not incident to an adjacency, and so for clerical purposes, we say that *v* has the null adjacency {*v*,*∅*}. A genome consisting of only circular (linear) chromosomes is called a circular (linear) *genome*. Note that *π *is circular if and only if the edges of *a*(*π*) form a perfect matching on *V*(*π*).

Henceforth, we only consider genome pairs {*π*,*Γ*} such that g(π)∪g(Γ)=G. A workhorse data structure encoding the relationship between *π* and *Γ *is the breakpoint graph ([13]), denoted by B(*π*,*Γ*) and defined as the edge-disjoint union ^a ^of *a*(*π*) and *a*(*Γ*), where adjacencies of *Γ *will be colored red (Figure 1(b)). Observe that *B*(*π*,*Γ*) is also a collection of disjoint paths and cycles, which alternate between red and blue edges. The *length *of a connected component of B(*π*,*Γ*) is its total number of edges; we consider an isolated vertex in B(*π*,*Γ*) to be a path of length 0. The breakpoint graph is also the line graph of the adjacency graph, which was first defined in [9] and has also been used in rearrangement studies.

A *double cut and join* operation (DCJ) on *π *(introduced in [8]) *uses* one or two adjacencies of *π* via one of the following four operations to produce a new genome *π*^′^: 

1. {*v*,*w*},{*x*,*y*} →{*v*,*x*},{*w*,*y*} 

2. {*v*,*w*},{*x*,*∅*}→{*v*,*x*},{*w*,*∅*} 

3. {*v*,*∅*},{*w*,*∅*} →{*v*,*w*} 

4. {*v*,*w*} →{*v*,*∅*},{*w*,*∅*}

**Figure 2:**
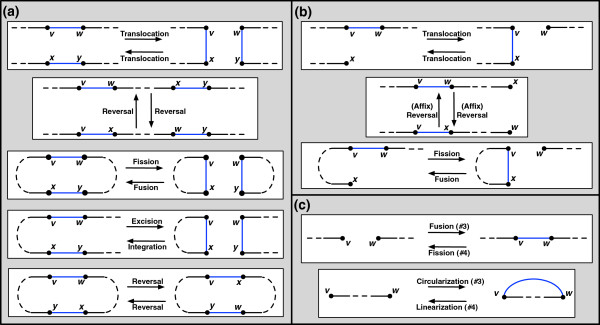
**The Collection of All Possible DCJ Operations. **The DCJ incorporates many operations, depending on the structure of the chromosomes involved and whether the adjacencies used belong to the same chromosome. **(a) **Operation 1 in the definition of the DCJ incorporates linear internal translocations, reversals, circular fusions/fissions, the excision of a circular chromosome from a linear chromosome, and the integration of a circular chromosome into a linear chromosome. **(b) **Operation 2 incorporates telomeric translocations, affix reversals (which involve the telomere of a linear chromosome), and the fission of a linear chromosome into a circular and linear chromosome (together with its inverse). **(c)** Operations 3 and 4 include linear fusions/fissions as well as the linearization/circularization of a single chromosome.

The DCJ incorporates a wide range of genome rearrangements, as shown in Figure 2.

For the particular case that *π *and *Γ *have the same genes (i.e., g(π)=g(Γ)=G), the *DCJ distance *between *π *and *Γ*, written *d*_DCJ_(*π*,*Γ*), is the minimum number of DCJs required to transform *π *into *Γ *One can easily verify that *d*_DCJ _forms a metric on the set of all genomes having gene set G. A closed formula for DCJ distance was derived in [9] and translated into breakpoint graph notation in [14]:

(1) $$d_{\text{DCJ}}(\pi ,\Gamma )=N-c(\pi ,\Gamma )-\frac{p_{\text{even}}(\pi ,\Gamma )}{2}$$

Here, *c*(*π*,*Γ*) and *p*_even_(*π*,*Γ*) denote the number of cycles and even-length paths in B(*π*,*Γ*), respectively.

For the more general case that *π *and *Γ *do not share the same genes, a deletion of a chromosomal interval of *π *replaces adjacencies {*v*,*w*} and {*x*,*y*} (contained in the order (*v*,*w*,*x*,*y*) along a chromosome of *π*) with the adjacency {*v*,*y*} and removes the path connecting *w *to *x*. We also allow deletions of entire chromosomes; however, we must stipulate (following the lead of the authors in [11]) that every vertex removed from *π *must belong to V-V(Γ). ^b ^The insertion of a chromosome or chromosomal interval into *π *to obtain *π*^′ ^is defined as the inverse of a corresponding deletion from *π*^′ ^that yields *π*. Note that a consequence of this definition is that we may not insert a gene unless it is contained in G. Insertions and deletions are collectively called indels; thus, we define the DCJ-indel distance between *π *and *Γ*, written $d_{\text{DCJ}}^{\text{ind}}(\pi ,\Gamma )$, as the minimum number of DCJs and indels required to transform *π* into *Γ*.

Because insertions and deletions are inverse operations, it follows that $d_{\text{DCJ}}^{\text{ind}}(\pi ,\Gamma )=d_{\text{DCJ}}^{\text{ind}}(\Gamma ,\pi )$. However, although $d_{\text{DCJ}}^{\text{ind}}$ is symmetric, unlike *d*_DCJ _it does not form a metric, as the triangle inequality does not hold; see [15] for a more complete discussion.

### DCJ-Indel sorting

#### Handling circular singletons

We begin our discussion of DCJ-indel sorting by defining a circular singleton of *π *(adapted from[11]) as a circular chromosome *C *such that *V*(*C*) ∩ *V* (*Γ*) = *∅*. Note that *C *is defined with respect to *Γ* as well as *π*. Ideally, we could delete (insert) all circular singletons of *π *and *Γ *immediately to simplify the problem of DCJ-indel sorting; fortunately, this is indeed the case, as shown by the following two results.

##### Proposition 1

*If *π^′^*is formed by removing a circular singleton C from *π,*then*$d_{\text{DCJ}}^{\text{ind}}(\pi^{\prime },\Gamma )=d_{\text{DCJ}}^{\text{ind}}(\pi ,\Gamma )-1$.*Furthermore, when transforming *π *into *Γ *via a minimum collection of DCJs and indels, no gene belonging to a circular singleton of *π *can ever appear in the same chromosome as a gene of * Γ.

##### Proof

Any collection of *k *DCJs and indels transforming *π*^′ ^into *Γ *can be supplemented by the deletion of *C* to yield *k *+ 1 DCJs and indels transforming *π *into *Γ*; thus, $d_{\text{DCJ}}^{\text{ind}}(\pi^{\prime },\Gamma )\geq d_{\text{DCJ}}^{\text{ind}}(\pi ,\Gamma )-1$.

To obtain the reverse bound, let us view a transformation T of *π *into *Γ *as a sequence (*π*_0_,*π*_1_,…,*π*_*n*_) (*n *≥ 1), where *π*_0 _= *π*, *π*_*n *_= *Γ*, and *π*_*i *+ 1 _is obtained from *π*_*i *_as the result of a single DCJ or indel. Consider the sequence $\left(\pi_{0}^{\prime },\pi_{1}^{\prime },\ldots ,\pi_{n}^{\prime }\right)$, where $\pi_{i}^{\prime }$ is constructed from *π*_*i *_by removing the subgraph of *π*_*i *_induced by the vertices of *C *under the stipulation that whenever we remove a path *P *connecting *v* to *w*, we replace adjacencies {*v*,*x*} and {*w*,*y*} in *π *with {*x*,*y*} in $\pi_{i}^{\prime }$. It is easy to see that $\pi_{0}^{\prime }=\pi^{\prime }$, $\pi_{n}^{\prime }=\Gamma$, and for every *i *in range, either $\pi_{i+1}^{\prime }$ is the result of a DCJ or indel applied to $\pi_{i}^{\prime }$ or $\pi_{i+1}^{\prime }=\pi_{i}^{\prime }$; thus, $\left(\pi_{0}^{\prime },\pi_{1}^{\prime },\ldots ,\pi_{n}^{\prime }\right)$ encodes a transformation of *π*^′ ^into *Γ *using at most *n *DCJs and indels. Furthermore, one can verify that $\pi_{i+1}^{\prime }=\pi_{i}^{\prime }$ only when an adjacency of *C *is used by a DCJ in T changing *π*_*i *_to *π*_*i *+ 1 _or when *π*_*i *+ 1 _is produced from *π*_*i *_by a deletion of vertices that all belong to *C*. At least one such operation must always occur in T; hence, $d_{\text{DCJ}}^{\text{ind}}(\pi^{\prime },\Gamma )\leq d_{\text{DCJ}}^{\text{ind}}(\pi ,\Gamma )-1$.

The proposition’s second conclusion follows from the fact that if for some *j *(1 ≤ *j *≤ *n *- 1), a chromosome of *π*_*j *_contains a gene *g*_1 _of *π *and a gene *g*_2 _of *C*, then one DCJ was required to combine *g*_1 _and *g*_2 _into the same chromosome, and another will be needed to separate them, yielding two distinct values of *i *for which $\pi_{i+1}^{\prime }=\pi_{i}^{\prime }$. From the first part of the proof, we may conclude that $d_{\text{DCJ}}^{\text{ind}}(\pi ,\Gamma )<n$. □ □

Letting sing (*π*,*Γ*) denote the total number of circular singletons of *π *and *Γ*, we have an immediate corollary.

##### Corollary 2

The DCJ-indel distance is given by the following:

(2) $$d_{\text{DCJ}}^{\text{ind}}(\pi ,\Gamma )=\text{sing}(\pi ,\Gamma )+d_{\text{DCJ}}^{\text{ind}}(\pi^{0},\Gamma^{0})$$

*where *π^0 ^(Γ^0^) *is formed by removing all circular singletons from *π (Γ).

With respect to DCJ-indel sorting, Corollary 2 allows us to assume without loss of generality that *π *and *Γ *do not contain any circular singletons.

**Figure 3:**
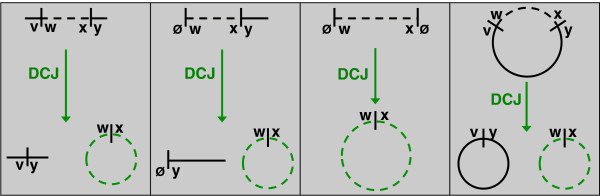
**Encoding the Deletion of a Chromosomal Interval as a DCJ. **The deletion of a chromosomal interval connecting *w *to *x *can be encoded by a DCJ that turns the interval connecting *w *to *x *into a circular chromosome. The four possible deletions of a chromosomal interval are shown in the above figure; this correspondence holds even when the interval in question is taken to be an entire linear chromosome.

We next make an observation taken from [16], which is that the deletion of a chromosomal interval of *π* connecting *w *to *x *may be viewed as a DCJ: {*v*,*w*},{*x*,*y *} → {*v*,*y*},{*w*,*x*}; this operation produces a circular chromosome containing *w *and *x *that is scheduled for removal, including the case that *v* or *y* equals *∅ *(the deletion of an entire linear chromosome is handled by *u *= *x *= *∅*); see Figure 3. Because insertions are the inverses of deletions, we would like to conclude that indels may be placed in a one-to-one correspondence with the removal of circular chromosomes. Ironically, the apparent exception to this proposed rule is the deletion of an entire circular chromosome.

Yet if a deleted circular chromosome *C *is not produced as the result of a DCJ, then *C *must be a circular singleton of *π *in order to be deleted. Otherwise, *C *has been produced as the result of a DCJ applied to a chromosomal interval; by the method we just described, we can amortize the deletion in this DCJ unless the DCJ also creates another circular chromosome *C*^′ ^that is scheduled for deletion. However, this sequence of operations cannot arise in a minimum collection of DCJs and indels transforming *π* into *Γ*, as we could simply delete the original chromosome from which *C* and *C*^′ ^were produced by the DCJ in question, thus requiring a single operation instead of three.

#### Toward a new model of Indels

We will follow the observation made in [16] that the actual removal of deleted chromosomes can occur as a final step in the transformation of *π *into *Γ*. As a result, we may view the transformation of *π *into *Γ *as composed of three steps: inserting chromosomes into *π *to yield a new genome *π*^′ ^with $g(\pi^{\prime })=G$; applying a sequence of DCJs to produce a genome *Γ*^′ ^having the same genes as *π*^′^; and finally, deleting chromosomes from *Γ*^′ ^to produce *Γ*. Note that we can equivalently view the first step as the deletion of chromosomes from *π*^′ ^to obtain *π*. Combining this observation with our correspondence between indels and circular chromosomes above, we may introduce the following framework.

Define a completion of *π *as a genome *π*^′ ^having $g(\pi^{\prime })=G$ and for which *a*(*π*^′^) is composed of *a*(*π*) together with a perfect matching on *V*(*π*^′^) - *V*(*π*). We call the adjacencies of *a*(*π*^′^) - *a*(*π*) new. Note that the chromosomes of *π *embed as chromosomes of *π*^′ ^and that the components of *π*^′ ^- *π *form cycles because the new adjacencies of *π*^′ ^induce a perfect matching on *V*(*π*^′^) - *V*(*π*); we may now without ambiguity call these circular chromosomes of *π*^′ ^the indels of *π*^′^. A completion of a pair of genomes (*π*,*Γ*) is simply a pair (*π*^′^,*Γ*^′^) for which *π*^′ ^and *Γ*^′ ^are completions of *π *and *Γ*, respectively. The above discussion implies that for any minimum cost transformation of *π* into *Γ*, the indels of *π*^′ ^correspond bijectively to DCJ operations, so that we will amortize each unit indel cost by that of a DCJ operation. This amortization yields the following equation for DCJ-indel distance:

(3) $$d_{\text{DCJ}}^{\text{ind}}(\pi ,\Gamma )=\underset{\left(\pi^{\prime },\Gamma^{\prime }\right)}{\min}\left\{d_{\text{DCJ}}(\pi^{\prime },\Gamma^{\prime })\right\}$$

where the minimum is taken over all completions of (*π*,*Γ*). A completion (*π*^∗^,*Γ*^∗^) is optimal if it attains the minimum in (3). Applying the closed form equation for the DCJ distance in (1) to immediately produces the following result.

##### Theorem 3

The DCJ-indel distance is given by the following equation:

(4) $$d_{\text{DCJ}}^{\text{ind}}(\pi ,\Gamma )=N-\underset{\left(\pi^{\prime },\Gamma^{\prime }\right)}{\max}\left\{c(\pi^{\prime },\Gamma^{\prime })+\frac{p_{\text{even}}(\pi^{\prime },\Gamma^{\prime })}{2}\right\}$$

*where the maximum is taken over all completions of *(π,Γ).

#### ***Constructing an optimal completion***

In light of Theorem 3, we have reduced DCJ-indel sorting to the problem of constructing indels intelligently to maximize a weighted sum of breakpoint graph components. Once we have produced an optimal completion (π^∗^,Γ^∗^), we can simply invoke the *O*(*N*) - time sorting algorithm described in [9] to transform *π*^∗ ^into *Γ*^∗ ^via a minimum collection of DCJs.

Our goal is to construct (*π*^∗^,*Γ*^∗^) by direct analysis of B(*π*,*Γ*). Because *π *and *Γ *do not necessarily share the same genes, B(*π*,*Γ*) may contain path endpoints that are not telomeres. Accordingly, we define a vertex *v *to be *Π *- open (*γ *- open) if *v *∉ *π *(*v *∉ *Γ*). In other words, *v *must be matched to some other *π *- open vertex when constructing the indels of *π*^∗^. ^c ^The paths of B(*π*,*Γ*) are therefore classified according to their endpoints: a *Π *- path (*γ *- path) ends in one *π *- open (*Γ *- open) vertex and one telomere (of either *π *or *Γ*); a *{Π*,*γ*} - path ends in a *π *- open vertex and a *Γ *- open vertex (such a path must have even length at least 2); a *{Π*,*Π*} - path (*{γ*,*γ*} - path) ends in two *π *- open (*Γ *- open) vertices and must therefore have odd length. We should also provide statistics for counting these different components. Define *p*^*Π*,*γ*^ as the number of {*π*,*Γ*} - paths in B(*π*,*Γ*); $p_{\text{even}}^{\Pi }$ as the number of even-length *π *- paths in B(*π*,*Γ*); and $p_{\text{even}}^{0}$ as the number of even-length paths in B(*π*,*Γ*) containing no open vertices (i.e., ending in two telomeres). Similar statistics counting odd-length paths can be defined analogously. We have dropped the genomes {*π*,*Γ*} from these statistics for the sake of simplicity; all component statistics will be taken with respect to B(*π*,*Γ*) unless otherwise noted.

We first present a proposition regarding the parity of the paths of B (π,Γ).

##### Proposition 4

*The component statistics of *B(π,Γ) *satisfy the following condition: *

(5) $$\begin{array}{ll} p^{\Pi ,\gamma }\equiv \left|p_{\text{odd}}^{\Pi }-p_{\text{even}}^{\Pi }\right|\;\equiv \left|p_{\text{odd}}^{\gamma }-p_{\text{even}}^{\gamma }\right|\;\mathrm{mod}\;2 & \; \end{array}$$

##### Proof

The total number of *π *- open vertices is equal to *V *(*π*^′ ^) - *V *(*π*) and must therefore be even. Of course, the same is the case for *Γ *- open vertices, and counting *π *- open and *Γ *- open vertices over the connected components of B (*π*,*Γ*) thus produces the following equivalences:

(6) $$\begin{array}{ll} p_{\text{odd}}^{\Pi }+p_{\text{even}}^{\Pi }+p^{\Pi ,\gamma } & \equiv 0\;\mathrm{mod}\;2\; \end{array}$$

(7) $$\begin{array}{ll} p_{\text{odd}}^{\gamma }+p_{\text{even}}^{\gamma }+p^{\Pi ,\gamma } & \equiv 0\;\mathrm{mod}\;2\; \end{array}$$

Adding *p*^*Π*,*γ *^to both sides of (6) and (7) gives the following:

(8) $$p^{\Pi ,\gamma }\equiv \left(p_{\text{odd}}^{\Pi }+p_{\text{even}}^{\Pi }\right)\;\equiv \left(p_{\text{odd}}^{\gamma }+p_{\text{even}}^{\gamma }\right)\;\;\mathrm{mod}\;2$$

The equivalence of (5) and (8) is an arithmetical fact. □

We next establish two necessary conditions on optimal completions by culling the set of possible adjacencies of any such completion. Our general strategy is to consider the addition of a new adjacency {*v*,*w*} to a completion *π*^′ ^as linking the component(s) of B(*π*,*Γ*) whose endpoints are the (*π *- open) vertices *v *and *w*. Our first result states that we must always link the endpoints of any {*Π*,*Π*} - path to each other.

##### Lemma 5

*If *(π^∗^,Γ^∗^) *is an optimal completion of *(π,*Γ*), then every {*Π*,*Π*} *- path (*{*γ*,*γ*} *- path) of length * 2 *k *- 1 *in* B(π,Γ)*(k *≥ 1*) * embeds into a cycle of length 2 *k *in B (π^∗^,*Γ*^∗^).

##### Proof

Let *P *be a path of length 2 *k *- 1 connecting *π*-open vertices *v *and *w *in B (*π*,*Γ*). Our claim is that we must link *v *and *w *in B (*π*^∗^,*Γ*^∗^). Suppose for the sake of contradiction that we have a completion (*π*^′^,*Γ*^′^) such that *P *does not embed into a cycle of length 2 *k *in B (*π*^′^,*Γ*^′^); in this case, we must have adjacencies {*v*,*x*} and {*w*,*y*} in *a*(*π*^′^), where all four vertices are distinct.

Consider the completion *π*^″ ^that is identical to *π*^′ ^except that {*v*,*x*} and {*w*,*y*} are replaced by {*v*,*w*} and {*x*,*y*}. In B(*π*^″^,*Γ*^′^), we have closed *P *into a cycle of length 2 *k*, and at the same time, we have changed neither the parity nor the linearity/circularity of the component containing *x *and *y*. Because we have increased the number of breakpoint graph cycles by 1 without changing the total number of paths, it follows from (1) that *d*_DCJ_(*π*^″^,*Γ*^′^) = *d*_DCJ_(*π*^′^,*Γ*^′^) - 1, and so (*π*^′^,*Γ*^′^) cannot be optimal. □

Having dealt with {*Π*,*Π*} - and {*γ*,*γ*} - paths of B(*π*,*Γ*), any remaining component of B(*π*^∗^,*Γ*^∗^) must be either a *j *- bracelet, which is a cycle linking *j *{*π*,*Γ*} - paths (where *j *≥ 2 and *j* is even), or a *k *- chain, in which two *π *- paths or two *Γ *- paths are linked via an intermediate number of {*π*,*Γ*} - paths to form a path containing *k *components from B(*π*,*Γ*) (*k *≥ 2). Note that when *k *is even, a *k*- chain *C *must contain either two *π *- paths or two *Γ*- paths, and when *k *is odd, *C *must contain one *π *- path and one *Γ *- path. 

For the sake of simplicity, we will represent a *j *- bracelet by (*P*_1 _: *P*_2 _: ⋯ : *P*_*j*_) and a *k *- chain by [*P*_1 _: *P*_2 _: ⋯ : *P*_*k*_], where every *P*_*i *_is linked to *P*_*i *+ 1_, and in the case of a *j *- bracelet, *P*_1 _is linked to *P*_*j*_. Because we wish to maximize a weighted sum of breakpoint graph components, we might guess that we should look for many short bracelets and chains. Indeed, the length of a bracelet or chain in B(*π*^∗^,*Γ*^∗^) is heavily restricted by the following lemma.

##### Lemma 6

*If *(π^∗^,Γ^∗^)*is an optimal completion, then a component C*^∗ ^*of *B (*π*^∗^,*Γ*^∗^) *can only contain two or more *{*π*,*Γ*} *- paths if C*^∗^*is a *2 *- bracelet.*

##### Proof

Again, say for the sake of contradiction that we have an optimal completion (*π*^′^,*Γ*^′^) for which a component *C*^′^ of B(*π*^′^,*Γ*^′^) contains two or more {*π*,*Γ*}-paths. If *C*^′ ^is not a 2 - bracelet, then it must contain {*π*,*Γ*} - paths *P*_1 _and *P*_2 _that are linked by precisely one new adjacency. Say that *P*_1 _joins *π *- open vertex *v *to *Γ *- open vertex *w *and that *P*_2 _joins *π *- open vertex *x *to *Γ *- open vertex *y*. To meet the assumption that *P*_1 _and *P*_2 _are linked by precisely one new adjacency, suppose that {*v*,*x*} ∈ *a*(*π*^′^) but {*w*,*y*} ∉ *a*(*Γ*^′^), where instead {*w*,*w*^′^} and {*y*,*y*^′^} are in *a*(*Γ*^′^). Replacing these two adjacencies with {*w*,*y*} and {*w*^′^,*y*^′^} defines a different completion *Γ*^″^ for which B(*π*^′^,*Γ*^″^) contains (*P*_1 _: *P*_2_). Viewed as an operation on B(*π*^′^,*Γ*^′^) to yield B(*π*^′^,*Γ*^″^), we have two cases.

First, if *C*^′^ was a bracelet, then we have formed two new bracelets from *C*^′^, one of which is (*P*_1 _: *P*_2_). Otherwise, *C*^′ ^was a chain, in which case we have formed a chain (of the same parity) in addition to (*P*_1 _: *P*_2_). In either case, *d*_DCJ _(*π*^′^,*Γ*^″^)<*d*_DCJ _(*π*^′^,*Γ*^′^), and so (*π*^′^,*Γ*^′^) cannot be optimal. □

Following Lemma 6, we may only have 2 - bracelets, 2 - chains, and 3 - chains in B (*π*^∗^,*Γ*^∗^). After a simple result about 2-chain components, we will be ready to state our main result on DCJ-indel sorting.

##### Proposition 7

The breakpoint graph of an optimal completion cannot have one 2-chain joining two odd π - paths and another 2-chain joining two even π-paths. The same holds for Γ - paths.

##### Proof

Once again, proceed by contradiction and assume that (*π*^′^,*Γ*^′^) is an optimal completion with such 2-chains [*P*_1 _: *P*_2_] and [*P*_3 _: *P*_4_]. Replacing these 2-chains with [*P*_1 _: *P*_3_] and [*P*_2 _: *P*_4_] replaces two odd paths in B(*π*^′^,*Γ*^′^) with two even paths; hence, (*π*^′^,*Γ*^′^) cannot be optimal. □

##### Theorem 8

*Algorithm *1, *given below, defines an O(N) time algorithm for DCJ - indel sorting. For pairs *{π,Γ} *having *sing (π,Γ) = 0,* the DCJ-indel distance is given by the following equation: *

(9) $$\begin{array}{lll} d_{\text{DCJ}}^{\text{ind}}(\pi ,\Gamma ) & =N-\left[\left(c+p^{\Pi ,\Pi }+p^{\gamma ,\gamma }+\left\lfloor \frac{p^{\Pi ,\gamma }}{2}\right\rfloor \right)\right)\; & \;\\ & \;+\frac{1}{2}\left(p_{\text{even}}^{0}+\min\left\{\overset{\Pi }{\underset{\text{odd}}{p}},p_{\text{even}}^{\Pi }\right\}\right)\; & \;\\ & \;\;\left(\left(+\min\left\{\overset{\gamma }{\underset{\text{odd}}{p}},p_{\text{even}}^{\gamma }\right\}+\delta \right)\right]\; \end{array}$$

Here, *δ *= 1 when *p*^*Π*,*γ *^is odd and either $p_{\text{odd}}^{\Pi }>p_{\text{even}}^{\Pi }$, $p_{\text{odd}}^{\gamma }>p_{\text{even}}^{\gamma }$ or $p_{\text{odd}}^{\Pi }<p_{\text{even}}^{\Pi },p_{\text{odd}}^{\gamma }<p_{\text{even}}^{\gamma }$; otherwise, *δ *= 0.

##### Proof

We aim to construct an optimal completion (*π*^∗^,*Γ*^∗^) having

(10) $$\begin{array}{ll} c(\pi^{*},\Gamma^{*}) & =c+p^{\Pi ,\Pi }+p^{\gamma ,\gamma }+\left\lfloor \frac{p^{\Pi ,\gamma }}{2}\right\rfloor \; \end{array}$$

(11) $$\begin{array}{lll} p_{\text{even}}(\pi^{*},\Gamma^{*}) & =p_{\text{even}}^{0}+\min\left\{\overset{\Pi }{\underset{\text{odd}}{p}},p_{\text{even}}^{\Pi }\right\}\; & \;\\ & \;+\min\left\{\overset{\gamma }{\underset{\text{odd}}{p}},p_{\text{even}}^{\gamma }\right\}+\delta \; \end{array}$$

First, we count the cycles of B(*π*^∗^,*Γ*^∗^). By Lemma 5, every {*Π*,*Π*} - path or {*γ*,*γ*} - path of B (*π*,*Γ*) must be closed into a cycle by adding a single new adjacency (Step 1 of Algorithm 1). We now claim that there exists an optimal completion containing $\left\lfloor \frac{p^{\Pi ,\gamma }}{2}\right\rfloor$ 2 - bracelets. Note that we may always replace 3-chains [*P*_1 _: *P*_2 _: *P*_3_] and [*P*_4 _: *P*_5 _: *P*_6_] (where *P*_1 _and *P*_4 _are *π *- paths) with [*P*_1 _: *P*_4_], (*P*_2 _: *P*_5_), and [*P*_3 _: *P*_6_], without increasing the DCJ distance of the associated completion because we have obtained a cycle from two paths. This argument implies Step 2 of Algorithm 1 and produces the value of *c*(*π*^∗^,*Γ*^∗^) stated above.

As for the even paths of B(*π*^∗^,*Γ*^∗^), let us operate under the assumption that *p*^*Π*,*γ*^ is odd. Then after forming a maximal collection of 2-bracelets, we will be left with one additional {*π*,*Γ*} - path *P*. We claim that (*π*^∗^,*Γ*^∗^) will be optimal if we link as many *π *- paths (*Γ *- paths) of opposite parity as possible. On the one hand, Proposition 7 states that we cannot have 2 - chains [*P*_1 _: *P*_2_] and [*P*_3 _: *P*_4_], where *P*_1 _and *P*_2 _are even *π *- paths and *P*_3 _and *P*_4 _are odd *π *- paths. On the other hand, say that we have a 2 - chain [*P*_1 _: *P*_2_] and a 3 - chain [*P*_3 _: *P *: *P*_4_], where without loss of generality we assume that *P*_1 _and *P*_2 _are odd *π *- paths, *P*_3 _is an even *π *- path, and *P*_4 _is a *Γ *- path. Replacing these chains with the chains [*P*_1 _: *P*_3_] and [*P*_2 _: *P *: *P*_4_] does not change the number of paths of even length in B(*π*^∗^,*Γ*^∗^), implying Step 3 of Algorithm 1

As a result, all remaining *π *- paths must have the same parity, as must all the *Γ *- paths; thus, we may choose any *π *- path and *Γ *- path to link to *P *(Step 4 of Algorithm 1) and form a 3-chain. The length of this 3-chain may be even (*δ *= 1) or odd (*δ *= 0) depending on whether the length of its *π *- path and *Γ *- path have equal parity or not. All remaining paths must therefore be 2-chains linking pairs of *π *- paths or pairs of *Γ *- paths (Step 5 of Algorithm 1).

If instead *p*^*Π*,*γ *^is even, then *δ *= 0, and the argument for constructing an optimal completion proceeds similarly, except that no {*π*,*Γ*} - paths will remain after forming a maximal collection of 2-bracelets, eliminating the need for Step 4. □

##### Algorithm 1.

Given genomes (*π*,*Γ*), the following algorithm constructs an optimal completion (*π*^∗^,*Γ*^∗^) in *O*(*N*) time. 

0 Remove all circular singletons from *π *and *Γ*. 

1 Close every {*Π*,*Π*} - path ({*γ*,*γ*}-path) into a cycle by adding a single new adjacency to *π*^∗^ (*Γ*^∗^). 

2 Form a maximum set of 2-bracelets. 

3 Form a maximum set of even 2 - chains by linking pairs of *π*-paths (*Γ *- paths) having opposite parity. 

4 If *p*^*Π*,*γ*^ is odd, then link the remaining {*π*,*Γ*} - path with any remaining *π *- path and *Γ *- path to form a 3 - chain. 

5 Arbitrarily link pairs of remaining *π *- paths, all of which have the same parity, to form 2-chains. Do the same for remaining *Γ*-paths.

### The solution space of DCJ-Indel sorting

The problem of DCJ sorting is well understood, its solution space having been described in [17]. Thus, by Theorem 3, to identify the solution space of DCJ-indel sorting (an open problem), we simply need to enumerate the construction of indels of an optimal completion. We mentioned this enumeration in [12], but here we will explore the details of the calculation.

#### Handling circular singletons

By Proposition 1, we may consider the circular singletons of *π *and *Γ *independently of other chromosomes; for that matter, because insertions and deletions are defined symmetrically, we may assume that *π *contains *k* chromosomes and that *Γ *is the empty genome. Then by Corollary 2 and the trivial fact that any DCJ applied to *π *changes the total number of chromosomes of *π *by at most 1 (see [8]), we may obtain *Γ *from *π *in *k *steps if and only if we perform *j *successive DCJs (0 ≤ *j *<*k*), each of which fuses two circular chromosomes into one, followed by applying *k *- *j *chromosome deletions.

Assuming that *k *is relatively small, the enumeration of all such transformations of *π *into *Γ* poses a tedious but straightforward task, as a fusion of two circular chromosomes corresponds to a DCJ using two adjacencies from different chromosomes.

#### Genomes lacking circular singletons

Having handled circular singletons, we may assume that sing(*π*,*Γ*) = 0. Fortunately, the lemmas presented before Theorem 8 have greatly reduced the collection of possible optimal completions, which we now continue to pare down.

##### Proposition 10

Every π - path (Γ - path) embedding into a 3-chain of an optimal completion must have the same parity.

##### Proof

Say for the sake of contradiction that we have an optimal completion (*π*^′^,*Γ*^′^) such that B(*π*^′^,*Γ*^′^) contains 3-chains [*P*_1 _: *P*_2 _: *P*_3_] and [*P*_4 _: *P*_5 _: *P*_6_], where *P*_1 _and *P*_4 _are *π *- paths of opposite parity. Consider the completion (*π*^″^,*Γ*^″^), which is defined by rejoining adjacencies of (*π*^′^,*Γ*^′^) to form [*P*_1 _: *P*_4_], (*P*_2 _: *P*_5_), and [*P*_3 _: *P*_6_] in B(*π*^″^,*Γ*^″^). The 2-chain [*P*_1 _: *P*_4_] must have even length, and (*P*_2 _: *P*_5_) is a cycle; thus, *d*_DCJ _(*π*^″^,*Γ*^″^) <*d*_DCJ _(*π*^′^,*Γ*^′^), and so (*π*^′^,*Γ*^′^) cannot be optimal. □

##### Proposition 11

*If p*^Π,γ^*is even, then the breakpoint graph of an optimal completion must contain a maximum set of even-length 2 - chains.*

##### Proof

We proceed by contradiction. Say that (*π*^′^,*Γ*^′^) is an optimal completion for which an odd *π *- path *P*_1 _and an even *π *- path *P*_2_ are contained in different components of B(*π*^′^,*Γ*^′^), neither of which is an even 2-chain. By Propositions 7 and 10, we may assume that *P*_1 _and *P*_2 _embed into an odd-length 2-chain [*P*_1 _: *P*_5_] and a 3-chain [*P*_2 _: *P*_3 _: *P*_4_]. Because *p*^*Π*,*γ*^ is even by Proposition 4, we must have at least one additional 3-chain [*P*_6 _: *P*_7 _: *P*_8_], where (again by Proposition 10) *P*_6_ is an even-length *π * -path, and the *Γ *- paths *P*_4 _and *P*_8 _have the same parity. With these assumptions in hand, we may rejoin adjacencies to form the four components [*P*_1 _: *P*_2_] (even), [*P*_5 _: *P*_6_] (even), (*P*_3 _: *P*_7_), and [*P*_4 _: *P*_8_] (odd), producing a cycle and two even 2-chains from our original three paths. Hence, by (4), (*π*^′^,*Γ*^′^) cannot be optimal. □

We are now ready to fully describe the collection of optimal completions when *p*^*Π*,*γ*^ is even. To construct an optimal completion, after closing each {*Π*,*Π*} - path and {*γ*,*γ*} - path, which can be done uniquely, we must form a maximum collection of even 2-chains by Proposition 11. Recall that our aim is to maximize the statistic $c(\pi^{*},\Gamma^{*})+\frac{p_{\text{even}}(\pi^{*},\Gamma^{*})}{2}$, and consider the following two subcases.

##### Case 1

*p*^*Π*,*γ *^is even, $p_{\text{odd}}^{\Pi }\leq p_{\text{even}}^{\Pi }$, and $p_{\text{odd}}^{\gamma }\geq p_{\text{even}}^{\gamma }$. First, a maximal collection of even-length 2 - chains will total $p_{\text{odd}}^{\Pi }+p_{\text{even}}^{\gamma }$ components, which requires simply choosing $p_{\text{odd}}^{\Pi }$ even-length *π *- paths, then matching them to odd-length *π *- paths. This can be achieved in *A*_1 _ways, where

(12) A1=pevenΠpoddΠ·poddΠ!=PpevenΠ,poddΠ

Next, we follow the same method for forming even-length 2-chains by linking *Γ *- paths of opposite parity, yielding *B*_1 _total matchings:

(13) $$B_{1}=P\left(p_{\text{odd}}^{\gamma },p_{\text{even}}^{\gamma }\right)$$

Here, we use P(*n*,*k*) to denote the partial permutation statistic: $P(n,k)=\frac{n!}{(n-k)!}$. We will be left with $p_{\text{even}}^{\Pi }-p_{\text{odd}}^{\Pi }$ even *π *- paths and $p_{\text{odd}}^{\gamma }-p_{\text{even}}^{\gamma }$ odd *Γ *- paths. It is impossible to create any more even-length paths in B(*π*^∗^,*Γ*^∗^), and so we must form a maximum collection of $\frac{p^{\Pi ,\gamma }}{2}$ 2 - bracelets from the {*π*,*Γ*} - paths:

(14) $$C_{1}=\left(p_{\text{even}}^{\Pi ,\gamma }-1\right)!!=\left(p_{\text{even}}^{\Pi ,\gamma }-1\right)\left(p_{\text{even}}^{\Pi ,\gamma }-3\right)\cdots (5)(3)(1)$$

Note the definition of double factorial. Finally, we link arbitrary remaining *π *- paths to each other and arbitrary remaining *Γ *- paths to each other:

(15) $$D_{1}=\left(p_{\text{even}}^{\Pi }-p_{\text{odd}}^{\Pi }-1\right)!!\cdot \left(p_{\text{odd}}^{\gamma }-p_{\text{even}}^{\gamma }-1\right)!!$$

By the independence of these four procedures, the total number of optimal completions is simply given by the product *A*_1 _· *B*_1 _· *C*_1 _· *D*_1_.

##### Case 2

*p*^*Π*,*γ *^is even, $p_{\text{odd}}^{\Pi }>p_{\text{even}}^{\Pi }$, and $p_{\text{odd}}^{\gamma }>p_{\text{even}}^{\gamma }$. In this case, we first form a maximum set of 2 - chains:

(16) $$A_{2}=P\left(p_{\text{odd}}^{\Pi },p_{\text{even}}^{\Pi }\right)\cdot P\left(p_{\text{odd}}^{\gamma },p_{\text{even}}^{\gamma }\right)$$

We then have $p_{\text{odd}}^{\Pi }-p_{\text{even}}^{\Pi }$ odd-length *π *- paths and $p_{\text{odd}}^{\gamma }-p_{\text{even}}^{\gamma }$ odd-length *Γ *- paths remaining. Assume without loss of generality that $p_{\text{odd}}^{\Pi }-p_{\text{even}}^{\Pi }\geq p_{\text{odd}}^{\gamma }-p_{\text{even}}^{\gamma }$, and set $m=\min\{p^{\Pi ,\gamma },p_{\text{odd}}^{\gamma }-p_{\text{even}}^{\gamma }\}$. We may attain the formula in (9) if and only if we form 2*j* even-length 3 - chains for some integer *j *satisfying $0\leq j\leq \frac{m}{2}$, then create $\frac{p^{\Pi ,\gamma }}{2}-j$ total 2 - bracelets from the remaining {*π*,*Γ*} - paths. Any remaining odd-length *π *- paths (*Γ *- paths) must then be linked to each other to form (odd-length) 2 - chains in B (*π*^∗^,*Γ*^∗^). The number of such possibilities can be counted by the following statistic *B*_2_:

(17) B2=∑j=0m/2poddΠ-pevenΠ2jpoddγ-pevenγ2jpΠ,γ2j(2j)!2·poddΠ-pevenΠ-2j-1!!poddγ-pevenγ-2j-1!!pΠ,γ-2j-1!!

Again, the two statistics can be carried out independently, yielding *A*_2 _· *B*_2 _total optimal completions.

In both of the first two cases, reversing the inequalities will lead to analogous arguments. For the next two cases, suppose instead that *p*^*Π*,*γ*^ is odd, and select a single {*π*,*Γ*} - path *P* that must belong to a 3 - chain.

##### Case 3

*p*^*Π*,*γ*^ is odd, $p_{\text{odd}}^{\Pi }<p_{\text{even}}^{\Pi }$, and $p_{\text{odd}}^{\gamma }>p_{\text{even}}^{\gamma }$. Note that there are *A*_3 _= *p*^*Π*,*γ*^ total ways to select a {*π*,*Γ*} - path *P*. Of the four possibilities for the parity of the paths to which *P *may be linked to form a 3 - chain, one may wish to verify that the only way we cannot attain the maximum in (9) is if we link *P *to an odd-length *π *- path and an even-length *Γ *- path. Thus, we arrive at three mutually exclusive subcases.

In our first subcase, *P *is linked to an even-length *π *- path and an odd-length *Γ *- path:

(18) $$B_{3}=p_{\text{even}}^{\Pi }\cdot p_{\text{odd}}^{\gamma }$$

We now have an even number of {*π*,*Γ*} - paths remaining and have reduced our problem to a simpler one that falls under Case 1 above, from which we may obtain some number *C*_3 _of optimal completions.

In the second subcase, we join *P* to an odd-length *π *- path and an odd-length *Γ *- path. First, select two such paths:

(19) $$D_{3}=p_{\text{odd}}^{\Pi }\cdot p_{\text{odd}}^{\gamma }$$

Again we have reduced the problem to a subproblem falling under Case 1, from which we may obtain *E*_3 _total optimal completions. In our third and final subcase, we join *P* to an even *π *- path and an even *Γ *- path:

(20) $$F_{3}=p_{\text{even}}^{\Pi }\cdot p_{\text{even}}^{\gamma }$$

Say that applying Case 1 to the resulting subcase in which *p*^*Π*,*γ*^ is even yields *G*_3 _total optimal completions. Then by independence, the total number of optimal completions over all three subcases will be given by *A*_3 _· (*B*_3 _· *C*_3 _+ *D*_3 _· *E*_3 _+ *F*_3 _· *G*_3_).

##### Case 4

*p*^*Π*,*γ *^is odd, $p_{\text{odd}}^{\Pi }>p_{\text{even}}^{\Pi }$, and $p_{\text{odd}}^{\gamma }>p_{\text{even}}^{\gamma }$. Having selected *P *from the *A*_4 _= *p*^*Π*,*γ*^ total {*π*,*Γ*} - paths, one may verify that the only way we can achieve the maximum in (9) is by linking *P *to an odd-length *π *- path and an odd-length *Γ *- path, of which there are $B_{4}=p_{\text{odd}}^{\Pi }\cdot p_{\text{odd}}^{\gamma }$ total choices. We have therefore reduced our problem of linking components of B(*π*,*Γ*) to a smaller problem, falling under Case 2, for which *p*^*Π*,*γ *^is even. If there are *C*_4 _total solutions to this smaller problem, then the number of optimal completions is given by *A*_4 _· *B*_4 _· *C*_4_.

As in the first two cases, reversing the inequalities defining Cases 3 and 4 will result in analogous arguments.

## Conclusions

In this paper, we have demonstrated how the problem of DCJ-indel sorting, first solved in [11], can equally be handled via direct inspection of the breakpoint graph. Unfortunately, we still do not see a natural correspondence between the two approaches to DCJ-indel sorting, which appear to be at odds because their definitions of indels are equivalent but motivated differently.

Furthermore, modeling an indel as a circular chromosome resulting from a DCJ has uncovered the solution space of DCJ-indel sorting, thus resolving an open problem. We wonder if other operations could be adapted to a similar model to yield a straightforward calculation of other genomic distances involving indels. We are also curious whether this model applies to the case of finding a minimum-cost transformation of one genome into another as we vary the parameter associated with the (constant) indel cost.

## Endnotes

^a^This definition allows B(*π*,*Γ*) to contain cycles of length 2.^b^In particular, this requirement bars the trivial transformation of *π *into *Γ *in which every chromosome from *π *is deleted, and then all the chromosomes of *Γ *inserted. ^c^Note that *v *cannot be simultaneously *π *- and *Γ *- open, although it may be a telomere of both *π *and *Γ *or be *π *- open and a telomere of *Γ *(in both cases, *v *is an isolated vertex of B(*π*,*Γ*), i.e., a path of length 0).

## Competing interests

The author declares that he has no competing interests.
